# Glucocorticoids Inhibit EGFR Signaling Activation in Podocytes in Anti-GBM Crescentic Glomerulonephritis

**DOI:** 10.3389/fmed.2022.697443

**Published:** 2022-02-10

**Authors:** Xiaomei Wu, Lu Ren, Qianqian Yang, Hui Song, Qiaoli Tang, Mingchao Zhang, Jiong Zhang, Zheng Tang, Shaolin Shi

**Affiliations:** ^1^National Clinical Research Center for Kidney Diseases, Jingling Hospital, Nanjing University School of Medicine, Nanjing, China; ^2^National Clinical Research Center of Kidney Diseases, Jinling Clinical Medical College of Nanjing Medical University, Nanjing, China

**Keywords:** anti-GBM glomerulonephritis, glucocorticoids, EGFR/STAT3 signaling, Notch signaling, podocyte, parietal epithelial cell

## Abstract

Glucocorticoids are commonly used to treat anti-GBM crescentic glomerulonephritis, however, the mechanism underlying its therapeutic effectiveness is not completely understood. Since podocyte EGFR/STAT3 signaling is known to mediate the development of anti-GBM glomerulonephritis, we investigated the effect of glucocorticoids on EGFR/STAT3 signaling in podocytes. We found that the levels of phosphorylated (activated) EGFR and STAT3 in podocytes were markedly elevated in anti-GBM patients without glucocorticoids treatment, but were normalized in patients with glucocorticoids treatment. In a rat model of anti-GBM glomerulonephritis, glucocorticoids treatment significantly attenuated the proteinuria, crescent formation, parietal epithelial cell (PEC) activation and proliferation, accompanied by elimination of podocyte EGFR/STAT3 signaling activation. In cultured podocytes, glucocorticoids were found to inhibit HB-EGF-induced EGFR and STAT3 activation. The conditioned medium from podocytes treated with HB-EGF in the absence but not presence of glucocorticoids was capable of activating Notch signaling (which is known to be involved in PEC proliferation and crescent formation) and enhancing proliferative activity in primary PECs, suggesting that glucocorticoids prevent podocytes from producing secreted factors that cause PEC proliferation and crescent formation. Furthermore, we found that glucocorticoids can downregulate the expression of EGFR ligands, EGF and HB-EGF, while upregulate the expression of EGFR inhibitor, Gene 33, explaining how glucocorticoids suppress EGFR signaling. Taken together, glucocorticoids exert therapeutic effect on anti-GBM crescentic glomerulonephritis through inhibiting podocyte EGFR/STAT3 signaling and the downstream pathway that leads to PEC proliferation and crescent formation.

## Introduction

Anti-glomerular basement membrane (GBM) glomerulonephritis is a disease caused by autoantibodies that target collagen and are deposited on GBM. This disease manifests with rapid progression of glomerulonephritis and often reaches end-stage renal disease (ESRD) in a short time period. Treatment with glucocorticoids and other agents is effective and routinely used for the disease.

Pathologically, this disease manifests with crescent formation in glomeruli and sclerosis of glomeruli. Previous studies have shown that parietal epithelial cells (PECs) are the predominant cell type at the time when the Bowman's capsule is intact; while the Bowman's capsule is broken, several types of immune cells, including mononuclear macrophages, T lymphocytes and fibroblasts, are present as part of the crescents (1–3). Therefore, the activation and proliferation of PECs are essential for the development of crescents (4–6). Previous studies have demonstrated that podocytes are also involved in the formation of crescents (7–10). In addition to the presence of podocytes themselves in crescents, it has been suggested that podocytes that are exposed to immune deposits or inflammatory cytokines could secrete factors which promote proliferation of PECs (11–14), and several signaling pathways have been implicated in the process, including EGF signaling.

Epidermal growth factor receptor (EGFR) is a member of ErbB receptors family. Upon activation by EGF ligands (EGF, HB-EGF, etc.), EGFR is phosphorylated and subsequently activates a wide variety of intracellular signaling pathways which are essential for many cellular functions, such as proliferation, migration, differentiation, and apoptosis, etc. (15–17). In a previous study, Bollée G and colleagues showed that the phosphorylation of EGFR in podocytes by autocrine heparin-binding EGF (HB-EGF) resulted in activation of PECs and development of crescents in a mouse model of anti-GBM disease. Deletion of the *EGFR* gene in podocytes of mice alleviated crescent formation, and pharmacological blockade of EGFR was also effective even after massive formation of crescents (18). In another study, deletion of signal transducer and activator of transcription 3 (STAT3) a downstream target of activated EGFR, in podocytes was found to markedly reduce crescent formation in the mouse model of anti-GBM glomerulonephritis (19). Thus, EGFR/STAT3 signaling pathway plays a critical role in the formation of crescents.

Glucocorticoids are commonly used for the treatment of anti-GBM crescentic glomerulonephritis (20), however, the mechanism underlying the therapeutic effectiveness of glucocorticoids on the disease is elusive. In the present study, we aim to uncover the mechanisms by determining the effect of glucocorticoids on EGFR/STAT3 pathway. Our previous work has confirmed the therapeutic effectiveness of glucocorticoids on anti-GBM crescentic glomerulonephritis as shown by the improved clinical and pathological parameters and attenuated PEC proliferation and crescent formation in patients with anti-GBM nephritis (21). In the present study, we examined the expression of EGFR, and STAT3 in the glomeruli of the patients with and without glucocorticoid treatment, and further explored the molecular processes using *in vivo* and *in vitro* models.

## Methods

### Human Kidney Samples

A total of 34 patients with biopsy-proven anti-GBM nephritis were recruited at our center. Kidney biopsies from patients and normal kidney tissues from nephrectomy patients were obtained. The patients were divided into two groups: group 1 treated with glucocorticoids before renal biopsy (*n* = 22) and group 2 treated without glucocorticoids (*n* = 12). Para-carcinoma kidney tissues were used as control. Patients received glucocorticoid therapy with prednisone or methylprednisolone. Samples were from Renal Biobank of National Clinical Research Center of Kidney Diseases, Jiangsu Biobank of Clinical Resources. The protocol concerning the use of the patients' samples was approved by the Human Subjects Committee of Jinling Hospital, and informed consent was obtained from all the participants.

### Induction of Anti-GBM Crescentic Glomerulonephritis in Rats

Nephrotoxic serum (NTS) was prepared by immunizing New Zealand White rabbits with rat GBM as described previously (22). Wistar rats, aged 8–12 weeks and weighing 120–150 g, were sensitized with intraperitoneal injection of 0.5 mg rabbit IgG with complete Freund's adjuvant or normal saline as control. Seven days later, rats were injected with either 100 μl of NTS or normal rabbit serum alone as a control *via* the tail vein. All animal experiments were conducted following the Institute Animal Care and Use Committee of Jingling Hospital.

### Animal Treatments

Wistar rats injected with NTS were randomly divided into six groups: Group 1 (*n* = 5), receiving daily intraperitoneal injections of methylprednisolone (2.5 μg/g; Pfizer) on day 1 (24 h after the injection of NTS) through 7; Group 2 (*n* = 5), daily treatment of methylprednisolone (2.5 μg/g; Pfizer) and RU486 (20 μg/g; Sigma-Aldrich) on day 1 through 7; Group 3 (*n* = 5), daily treatment of saline intraperitoneally as control; Group 4 (*n* = 5), daily intraperitoneal injections of methylprednisolone (2.5 μg/g; Pfizer) on day 7 through 13; Group 5 (*n* = 5), daily treatment of methylprednisolone (2.5 μg/g; Pfizer) and RU486 (20 μg/g; Sigma-Aldrich) on day 7 through 13; and Group 6 (*n* = 5), daily treatment of saline intraperitoneally as control. Twenty-four hour after the final treatment, all rats were sacrificed and serum samples collected. Kidney samples were processed for histological analysis, preparation of protein, total RNA, and glomeruli. Methylprednisolone is a systemic glucocorticoid that is used to treat patients with glomerulonephritis and rats of NTS model in literature.

### Proteinuria and BUN Measurements

Twenty-four-hour urine samples were collected at day 1, 3, 7, 9, and 13. Twenty-four-hour proteinuria was measured using Bradford Kit (Beyotime, Shanghai) according to the manufacturer's instructions. BUN levels were measured with the QuantiChrom Creatinine Assay Kit (BioAssay Systems, Hayward, CA).

### Antibodies and Reagents

Primary antibodies, including the p-STAT3 monoclonal antibody (anti-Tyr705) (1:200 dilution, Cell Signaling Technology, #4113), the p-EGFR monoclonal antibody (anti-Tyr1068) (1:200 dilution, Cell Signaling Technology, #2234), the PAX-2 polyclonal antibody (1:200 dilution, Invitrogen, #71-60000), the CD44 monoclonal antibody (1:200 dilution, Abcam, ab16728), and the Notch1 poly polyclonal antibody (1:200, Wanlei biology, wl03097) were used to detect renal p-STAT3, p-EGFR, PECs, and activated PECs, respectively. Antibodies against EGFR (1:100 dilution, Cell Signaling Technology, ##2232) and STAT3 (1:100 dilution, Proteintech, 10253-2-AP) were used for IHC to detect total EGFR and STAT3 in kidney. Secondary antibody was the goat anti-rabbit IgG H and L (FITC) (1:200 dilution, Abcam, ab6717).

### Immunohistochemistry and Immunofluorescence

Immunohistochemical staining was routinely processed. Sections were deparaffinized, rehydrated, incubated with hydrogen peroxide, and treated with microwave for antigen retrieval. Then, 10% fetal calf serum was added to block non-specific background. Subsequently, the sections were incubated with a primary antibody, followed by incubation with Envision reagent (Dako) and 3, 3′-diaminobenzidine tetrahydrochloride (DAB) as the chromogen. Then, the sections were counterstained with hematoxylin. Normal rabbit serum was used as a negative control for the primary antibody. Image pro plus version 7.0 system (Media Cybernetics, Silver Spring, USA) was used for semi-quantification of immunohistochemistry staining.

Immunofluorescence experiments in rats were performed as follows. Frozen tissue sections were fixed in Paraformaldehyde for 5 min, blocked with 5% BSA for 30 min, and incubated with goat anti-rabbit IgG H and L (FITC).

### Podocyte Cell Line Culture

The cell line was the gift from Dr. Moin Saleem at Bristol University, UK (23). Podocytes were grown at 33°C and switched to and incubated at 37°C for 7–10 days. The cells were serum starved for overnight before treatment. The podocytes were treated with HB-EGF (1 ng/ml), HB-EGF (1 ng/ml) + Dexamethasone (Dex, 1 μM), respectively. Dex is a systemic glucocorticoid that is usually used for cultured cells.

### Glomerular Isolation and Primary Glomerular Parietal Epithelial Cell Culture

Glomeruli from Sprague-Dawley (SD) male rats that were 6-week-old and weighed ~200 g were isolated as previously described (24, 25). Briefly, renal cortex tissues were excised from kidney and cut into ~1 mm^3^ pieces. The tissue was then squashed through a 200-μm sieve and next passed a 70-μm sieve to retain the glomeruli on the sieve. The glomeruli were collected by gentle rinsing with cold PBS and aliquoted into dishes for culture. The percentages of capsulated glomeruli were about 20%. The isolated glomeruli were transferred onto a 6-well plate coated with type I collagen and cultured in RPMI 1640 medium supplemented with 10% FBS, 1% Pen/Strep and 1% ITS at 37°C. Glomeruli attached to the plate on day 5 of culture without any agitation. Three days later, candidate PECs were selected according to morphology, and glomeruli and other outgrowth of cells were removed from the plate. Cultured podocytes were treated with HB-EGF for 24 h, and the conditioned medium was collected and used to treat primary PECs for 24 h. The conditioned medium from cultured podocytes treated for 24 h was collected and added to the PECs. For a control that precluded any effect of HB-EGF in the conditioned medium on PECs concerning the molecules of interest, we added the same amount of HB-EGF in the control medium from the podocytes not treated with HB-EGF, and used it to treat PECs, together with the conditioned medium.

### Western Blot

Cells were lysed with RIPA buffer containing protease inhibitor cocktail (Roche, Indianapolis, IN) and phosphatase inhibitor. Protein concentrations of the supernatant samples were measured with BCA protein kit (Bio-Rad). The samples were mixed with loading buffer and boiled for 5 min. Ten percent of SDS-PAGE was used for electrophoresis of the samples, and the proteins were then transferred to membrane. The membrane was incubated with blocking solution containing 5% milk in TBST solution (20 mM Tris-HCl, PH 7.14, 150 mM NaCl, 0.1% Tween-20) at room temperature for 1 h, and then incubated with primary antibodies respectively, at 4°C overnight. The membrane was washed using TBST for 5 min (3X), and then incubated with HRP-labeled secondary antibody at room temperature for 1 h. After washed, the ECL system (Millipore) was used to detect the proteins. AlphaView (ProteinSimple, USA) was used to quantify band intensities on the blots.

### Real-Time Quantitative RT-PCR

Total RNA was extracted from isolated glomeruli, cultured podocytes or rat primary PECs using Mini Best Plant RNA Extraction Kit (Takara). The amount of RNA was determined using NanoDrop 1000 (Thermo Scientific, Rockford, IL). Total RNA was reverse transcribed using the PrimerScript RT Master Mix System (Takara) for first-strand cDNA synthesis. Quantitative RT-PCR was performed using Sybr Green Master Mix (Takara) and the Applied Biosystems 7900 Real-time PCR system. Ct values of the gene targets were normalized to GAPDH and 18s rRNA. The sequences of primers were shown in Supplementary Table S1. The fold change in the expression of target genes compared to the reference group was calculated using the 2^−ΔΔ^CT method.

### Statistical Analysis

Results are presented as means ± standard errors. Differences between two groups were analyzed with Student's *t*-tests. Comparisons between three or more groups were performed using the Kruskal–Wallis ANOVA, followed by least significant difference test. *P* < 0.05 was considered statistically significant.

## Results

### Glucocorticoids Treatment Inhibited EGFR/STAT3 Signaling Activated in Podocytes of the Patients With Anti-GBM Nephritis

**Figure 1:**
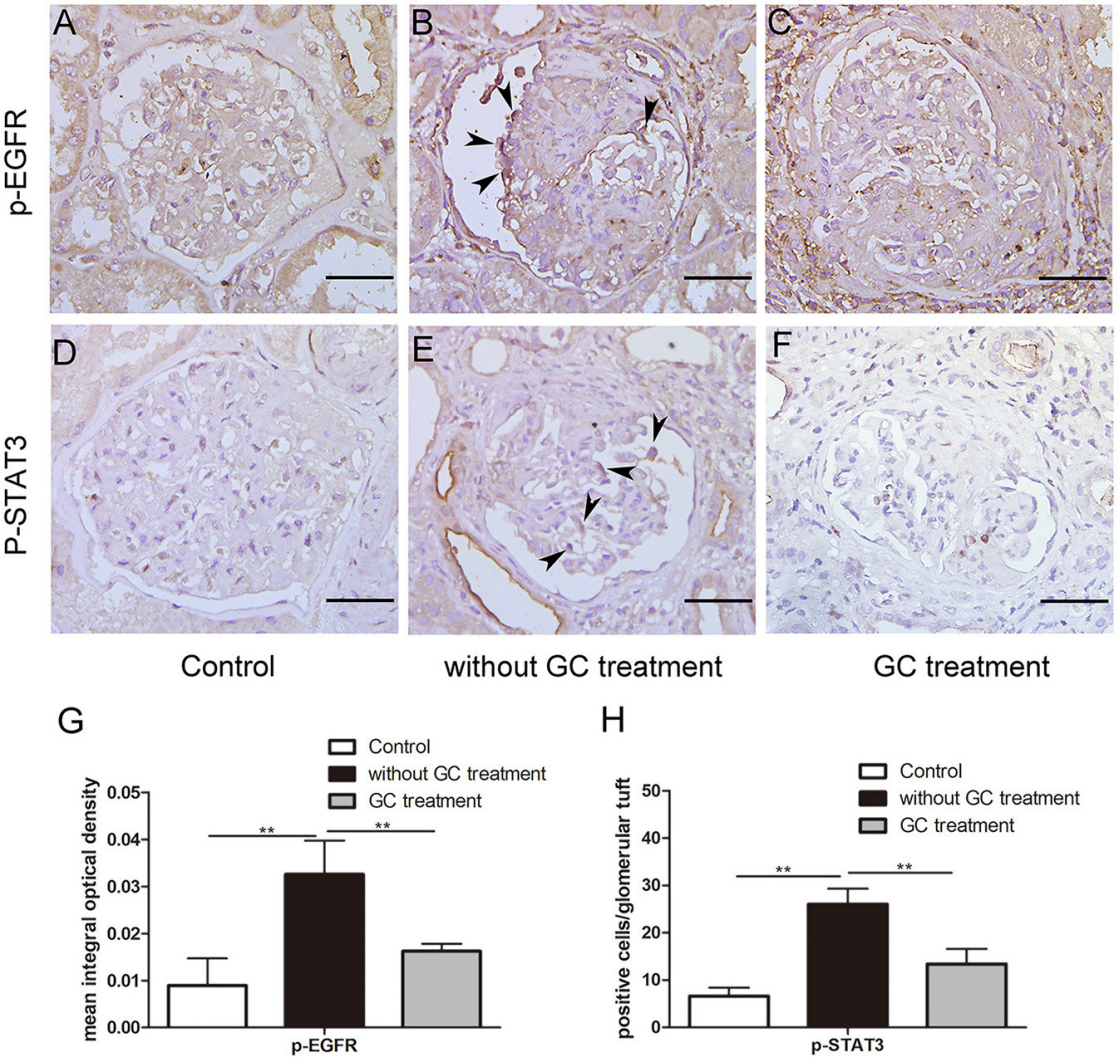
Glucocorticoids inhibited aberrant activation of EGFR-STAT3 signaling in podocytes of patients with anti-GBM nephritis. **(A–F)** Representative immunohistochemistry staining for p-EGFR and p-STAT3 in sections of kidney biopsies from patients with anti-GBM nephritis. Twenty-two patients who received glucocorticoid (GC) treatment before renal biopsy **(C,F)** and 12 patients without GC treatment before renal biopsy **(B,E)** were analyzed. Para-carcinoma kidney tissues **(A,D)** were used as control. **(G,H)** Quantification of intensity of IHC staining in glomeruli tuft for a-f. **P < 0.01 vs. the group without GC treatment. Scale bars, 50 μm.

To determine the effect of glucocorticoids on EGFR/STAT3 signaling in anti-GBM nephritis, we performed immunohistochemical staining of phosphorylated EGFR and STAT3 in the renal biopsies of the patients with (*n* = 22) and without (*n* = 12) glucocorticoids treatment. The information on the patients, including renal function, proteinuria, percent crescent formation and infiltrating immune cells in kidneys was showed in Table 1. Other details can be found in our previous work (21). As shown in Figure 1, immunostaining of p-EGFR or p-STAT3 was rarely seen in glomeruli of normal human kidney. In contrast, in patients with anti-GBM nephritis p-EGFR and p-STAT3 levels were both markedly elevated in the podocytes, which typically located at the periphery of the glomerular tuft. However, the elevation of p-EGFR and p-STAT3 levels was eliminated in podocytes of patients treated with glucocorticoids.

**Table 1:** Characteristics of the patients with anti-GBM (21).

	**GC treatment (*n* = 22)**	**Without GC treatment (*n* = 12)**	***P***
Age (years)	41.6 ± 17.7	39.7 ± 17.7	0.77
Duration (weeks)	8 (4–12)	3 (2–7)	0.39
Gender (female: male)	0.69	1	
Pulmonary hemorrhage (%)	4.5	0	
Gross hematuria (%)	63.6	58.3	1.0
Oliguria/anuria (%)	4.5	25.0	0.12
Urinary protein (mg/24 h)	3.0 ± 3.0	3.8 ± 3.2	0.48
SCr (mg/dl)	5.43 ± 3.20	9.66 ± 5.47	0.03^*^
Crescents (%)	75.2 ± 27.6	84.3 ± 17.1	0.25
Cellular crescents (%)	19.1 ± 19.4	52.4 ± 32.3	0.005^**^
Infiltrating cells (/mm^2^)	976 ± 393	1,077 ± 611	0.56

*SCr, serum creatinine; GBM, glomerular basement membrane; GC, glucocorticoid*;

* *p < 0.05*;

** *p < 0.01*.

### Generation of Rat Anti-GBM Crescentic Glomerulonephritis

**Figure 2:**
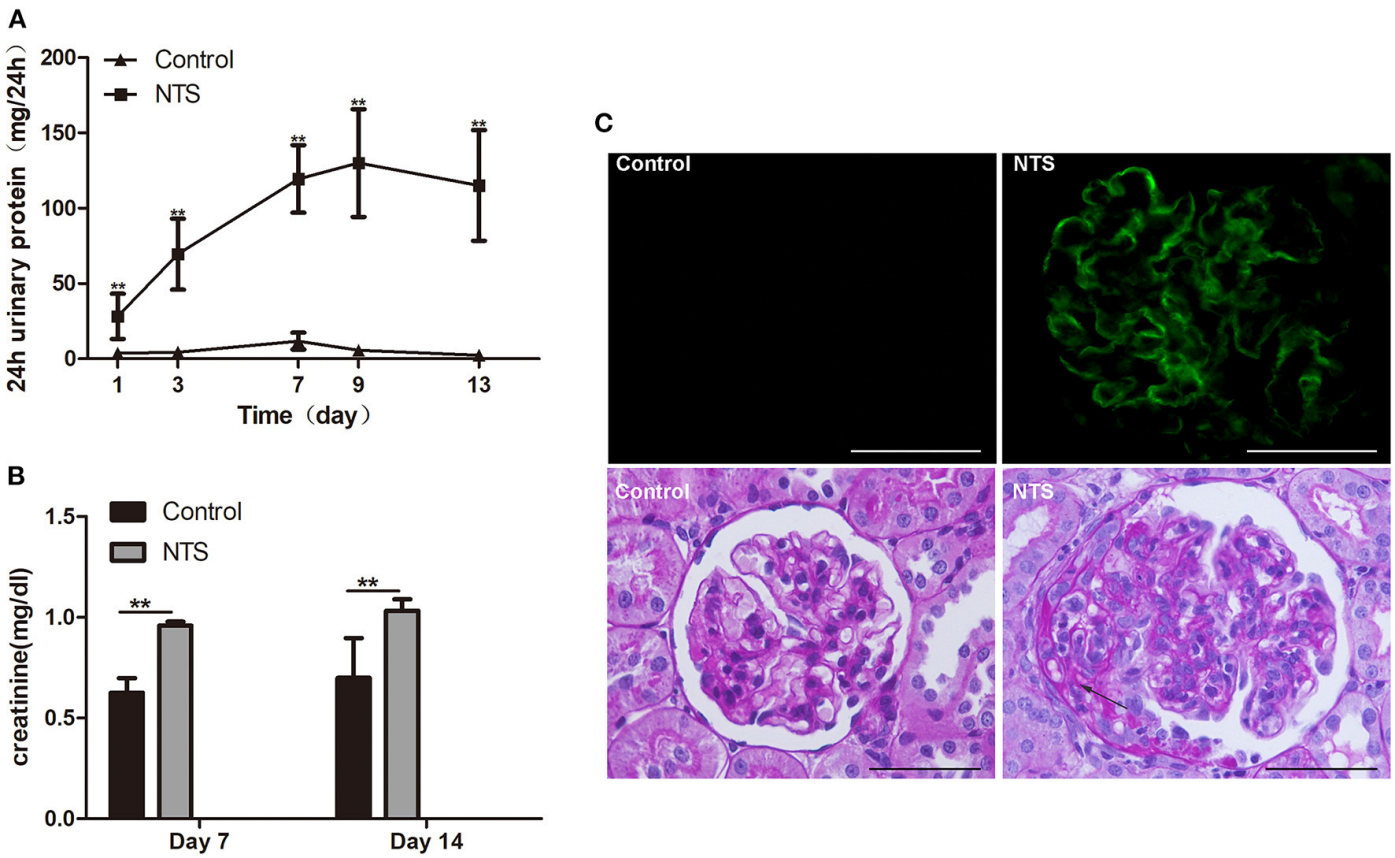
Clinicopathologically characteristics of rat model of anti-GBM nephritis. **(A)** 24-h urinary protein levels in rats after NTS injection and control (*n* = 5 per group). **(B)** Serum creatinine concentration of NTS and control rats at day 7 and day 14 (*n* = 5 per group). **(C)** Immunofluorescence staining of goat anti-rabbit IgG in kidney of NTS rats, showing strong linear deposition of IgG along GBM; and Periodic acid–Schiff staining of kidney sections at day 14, showing crescent formation (arrows) in glomeruli of NTS rats. **P < 0.01; Scale bars, 50 μm.

**Figure 3:**
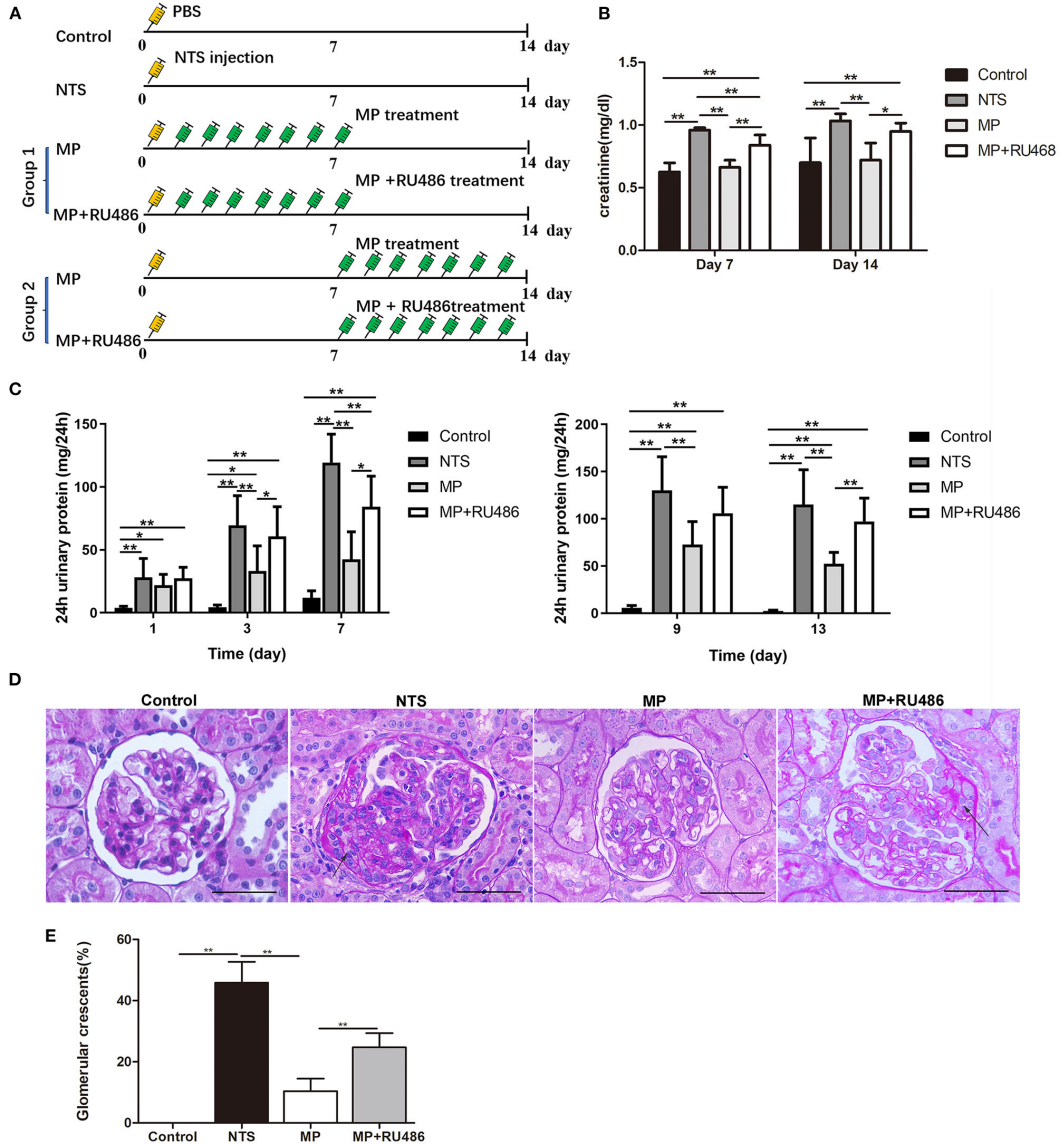
Glucocorticoid treatment attenuated the progression of rat anti-GBM glomerulonephritis. **(A)** Schematic of experimental design. **(B)** serum creatinine concentration of rats on day 7 and 14 after NTS injection (*n* = 5 per group). **(C)** 24-h urinary protein levels of rats on day 1, 3, 7, 9, and 13 after NTS injection (*n* = 5 per group). **(D)** Periodic acid–Schiff staining of kidney from rats 14 days after NTS injection showing crescent formation (arrows) in glomeruli. **(E)** The percentage of cellular crescents in rats.**P < 0.01, *P < 0.05 vs. controls. Scale bars, 50 μm.

To prove our hypothesis that glucocorticoids suppress crescent formation and glomerulonephritis through blocking EGF signaling in podocytes, we generated a rat model of accelerated anti-GBM nephritis. In this model, Wistar rats showed heavy proteinuria 24 h after injection of nephrotoxic serum (NTS), and the urinary protein levels continuously elevated and peaked at day 9 (Figure 2A). Meanwhile, the concentrations of serum creatinine in NTS-treated rats were also significantly increased at day 7 and day 14 compared to that of control rats (Figure 2B). Concerning the pathological changes, NTS rats showed a bright linear staining of IgG deposited on GBM on immunofluorescence as previously reported. Cellular crescents were evident at about day 7, and became diffused at day 14 when 45.9 ± 6.8% of the glomeruli were crescentic and most of the crescents occupied about 15 to 40% of the area of the glomeruli (Figures 2C, 3E).

### Glucocorticoids Treatment Attenuated the Progression of Anti-GBM Glomerulonephritis in Rats

We investigated the effectiveness of glucocorticoids (methylprednisolone, MP) on NTS-induced glomerulonephritis in rats following the scheme in Figure 3A. It was found that the progressive proteinuria of NTS-treated rats in the group, in which MP treatment started the next day (day 1) of NTS injection, was alleviated at day 3 and 7. MP was also effective even if the treatment began on day 7 when progressive proteinuria and crescent had developed (Figures 3C,D). Consistently, elevation of serum creatinine level of the NTS-treated rats was also attenuated significantly after consecutive administration of MP for 7 days (Figure 3B).

In addition to the clinical parameters, pathological changes caused by NTS were mostly prevented by MP treatment as shown by decreased percentage of crescentic glomeruli (10.4 ± 4.1% vs. 45.9 ± 6.8% prior to treatment), and decreased area of the crescents (<15% area of the glomeruli) (Figure 3E). We additionally found that administration of glucocorticoid receptor (GR) antagonist, RU486, markedly suppressed the therapeutic effectiveness of MP as shown by the proteinuria and crescent formation in the NTS rats that were treated with MP and RU486 for seven days, which were comparable to that of NTS rats without treatment (Figures 3C,D). This result indicated that MP exerted its effect through its receptor.

### Glucocorticoid Treatment Suppressed PEC Activation and Proliferation in NTS Rats

**Figure 4:**
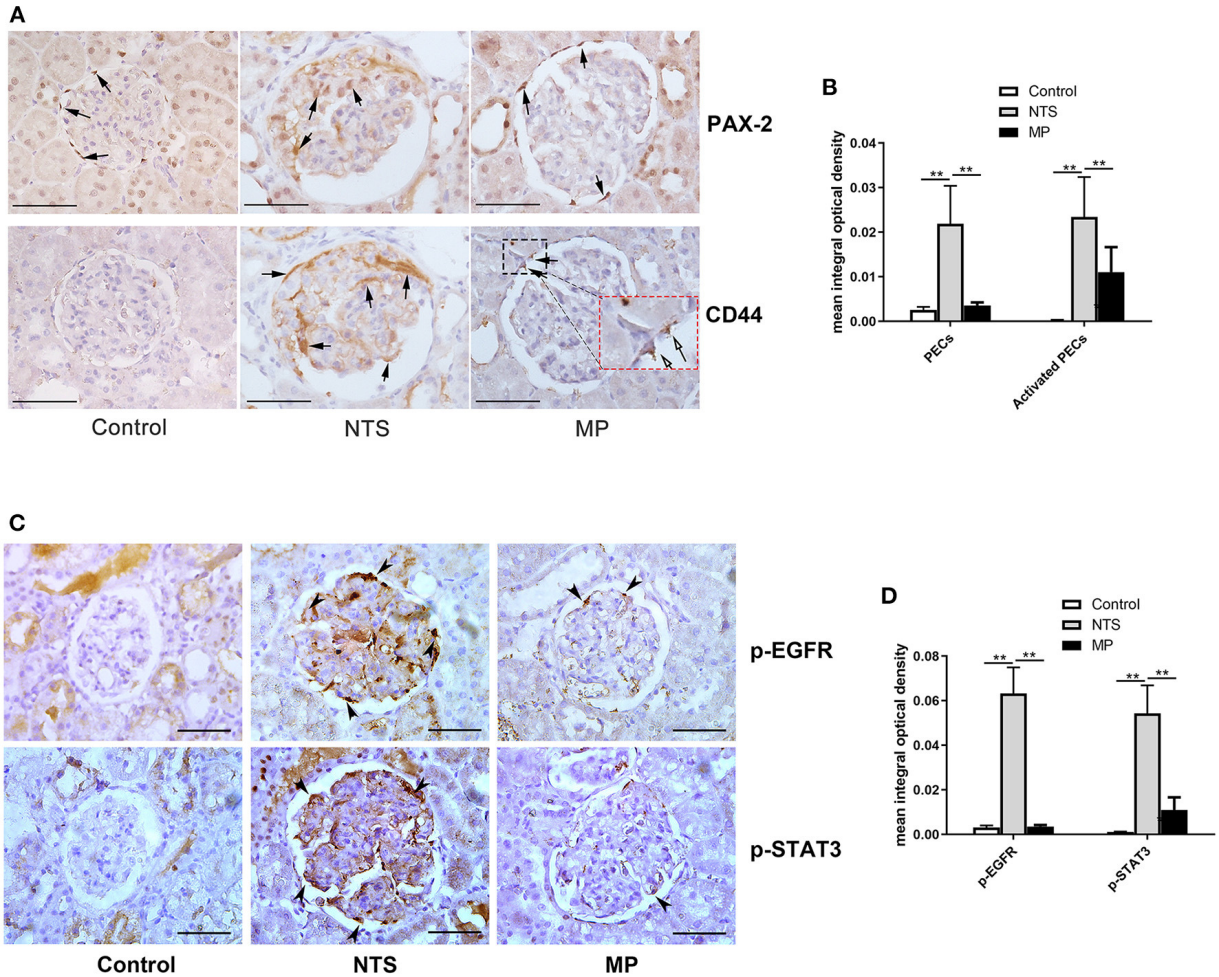
Glucocorticoid treatment suppressed proliferation of PECs and activation of EGF signaling in podocytes of NTS rats. **(A)** Immunohistochemistry staining for PAX-2 (PECs) and CD44 (activated PECs) in sections of kidney biopsies from rats treated with NTS alone, NTS+MP, and NTS+MP+RU486, respectively (*n* = 5 per group). Quantification of staining intensity in glomeruli is shown in **(B)**; **(C)** Immunohistochemistry staining for p-EGFR (Tyr1068) and p-STAT3 (Tyr705) in sections of kidney biopsies from the corresponding rats in **(A)**. Quantification of the staining intensity for p-EGFR and p-STAT3 in glomerular tuft is shown in **(D)**. Arrows denote positive staining in the glomeruli; **P < 0.01. Scale bars, 50 μm.

The activation and proliferation of PECs is an essential event in the formation of cellualr crescents. We found that the number of PAX-2 positive cells was markedly increased in the celluar crescents, as well as along the glomeular basement membrane of the rats 14 days after injection of NTS. These PAX-2 positive cells appeared to be CD44 positive as shown by immunohistochemistry staining on consecutive tissue sections showing colocalization of PAX-2 with CD44. CD44 is known to be *de novo* expressed in activated PECs in glomeruli (26). However, the numbers of proliferated and activated PECs were significantly reduced in the NTS rats after MP treatment from day 7 through 13, compared to untreated ones. Nevertheless, there was still some weak but consistent CD44 staining in glomeruli of the rats after MP treatment (Figures 4A,B). On the consecutive sections for PAX-2 and CD44 staining, we also observed a small portion of cells that were CD44 positive but PAX-2 negative. These cells were likely the activated mesangial cells or macrophages.

### Glucocorticiods Treatment Inhibited EGF Signaling in Podocyte of NTS Rats

As described earlier, we speculated that glucocorticoids treatment could suppress the proliferation of PECs and crescent formation by blocking EGF signaling in podocytes thus preventing podocytes to express secreted factors that promote proliferation of PECs. Previous studies have demonstrated that EGFR/STAT3 signaling pathway in podocytes is involved in the formation of crescents. Thus, we examined the activation of the components in the EGF signaling pathway by IHC staining in kidney of NTS rats. We found aberrant activation of EGFR and STAT3 in podocytes of the experimental glomerulonephritis model, as shown by the striking staining of phosphorylated EGFR and STAT3, which were not present in healthy controls and eliminated in the NTS rats with MP treatment (Figures 4C,D). In contrast, the total EGFR and STAT3 protein levels appeared not changed in the glomeruli (Supplemental Figure S1).

### Glucocorticoids Treatment Reduced Notch Signaling Pathway in Podocytes and PECs of NTS Rats

**Figure 5:**
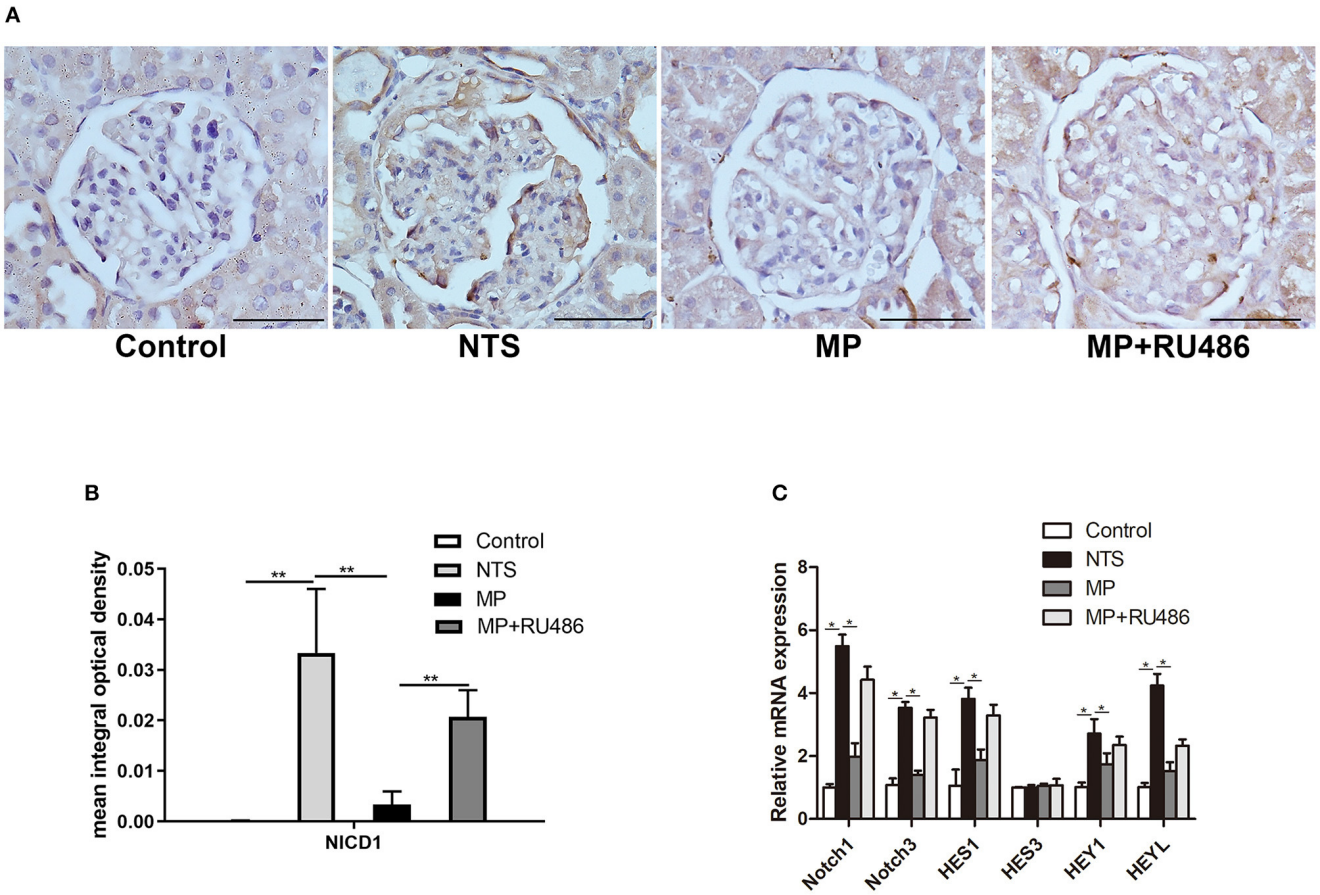
Glucocorticoids prevented Notch signaling activation in podocytes of NTS-treated rats. **(A)** Immunohistochemistry staining of NICD1 in sections of kidney biopsies from rats treated with saline (control), NTS, NTS+MP, and NTS+MP+RU486, respectively (*n* = 5 per group). Quantification of immunostaining intensity for NICD1 in glomerular tuft is shown in **(B)**; **(C)** The relative mRNA expression of Notch pathway genes (Notch1, Notch3, Hes1, Hes3, Hey1, and HeyL) from isolated glomeruli of the rats treated as indicated on day 14 (*n* = 5 per group). *P < 0.05, **P < 0.01. Scale bars, 50 μm.

Notch signaling is known to play a key role in the formation and progression of crescentic glomerulonephritis. In the present study, Notch family and their downstream transcriptional targets were examined. In kidney of control rats, Notch intracellular domain (NICD), a marker of Notch activation, was scarcely detected by IHC staining, but it was significantly upregulated in podocytes of the NTS rats (Figures 5A,B). Besides, quantitative reverse transcriptase–PCR (qRT–PCR) analysis with isolated glomeruli showed that expression of Notch-related genes (Notch1, Notch3, Hes1, Hey1, and HeyL) were significantly increased in glomeruli of the NTS rats compared with controls (Figure 5C). These results indicated that Notch signaling was activated in our experimental glomerulonephritis model. Previous studies have demonstrated that Notch signaling participates in the proliferation of PECs. Consistently, we observed NICD1 protein in PECs of the NTS rats (Figure 5A). Meanwhile, MP was found to prevent activation of Notch signaling as shown by the reduced NICD1 production (Figures 5A,B) and Notch target genes transcription (Figure 5C); as expected, GR antagonist RU486 suppressed these effects of MP (Figures 5A–C).

### Glucocorticoids Treatment Suppressed Activation of EGF and Notch Signaling Induced by HB-EGF in Cultured Podocytes

**Figure 6:**
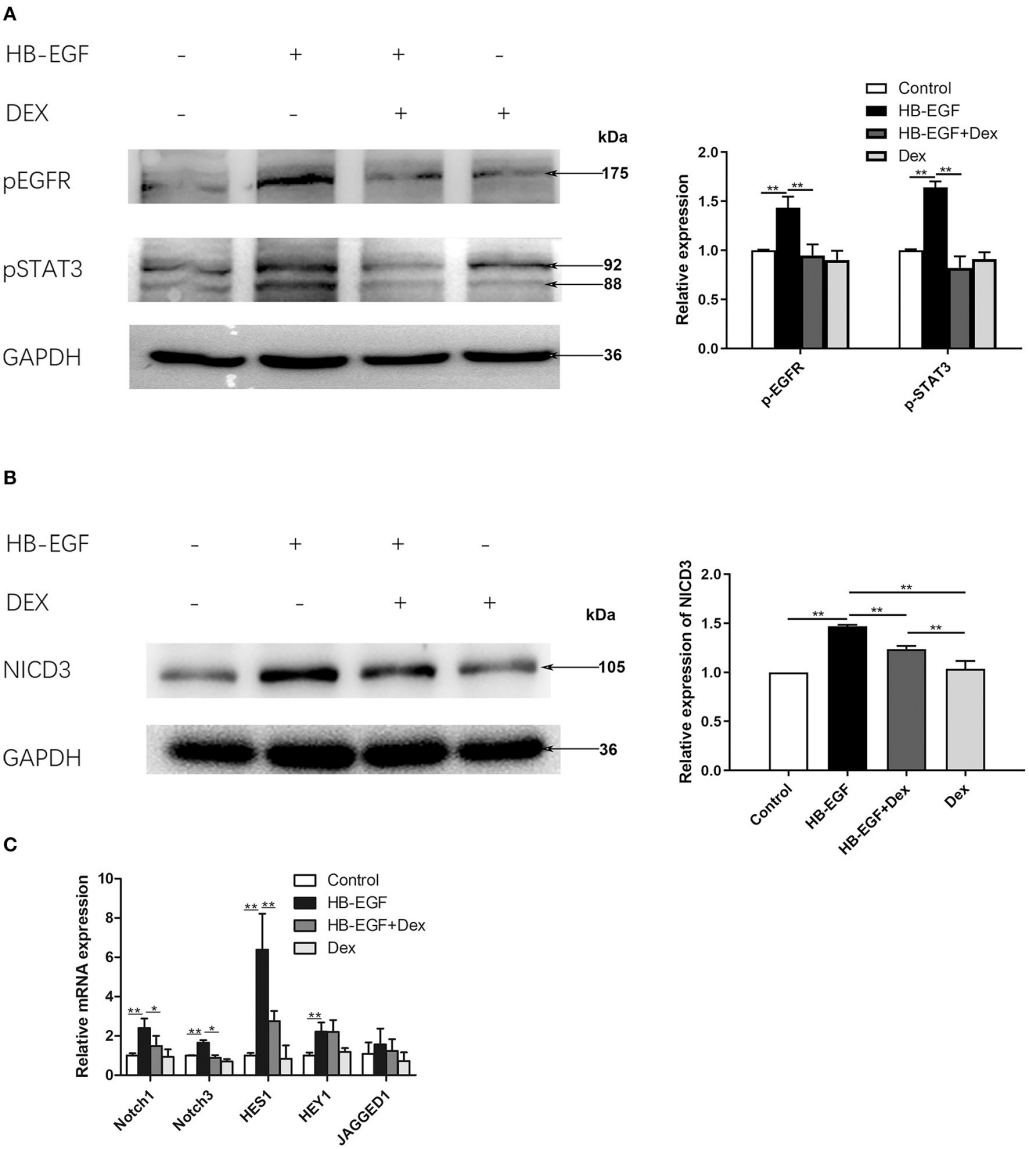
Glucocorticoid treatment suppressed activation of EGFR, STAT3 and Notch3 in HB-EGF-treated podocytes in culture. **(A)** Immunoblotting showing that treatment of Dex prevented phosphorylation of EGFR (Tyr1068) and STAT3 (Tyr705) in HB-EGF treated podocyte (target bands are indicated by arrows) according to the samples treated for 3 h. Quantifications of the blots are shown on the right. **(B,C)** Immunoblotting **(B)** and qPCR **(C)** showing Notch signaling was induced by HB-EGF, which was prevented by Dex. Quantification of the blots is shown on the right **(B)**. The data are expressed as the mean ± SD of three independent experiments **(C)**. *P < 0.05, **P < 0.01 vs. mRNA levels on 0 h at baseline.

To investigate the mechanism of how glucocorticoids inhibit EGF and Notch signaling, we performed *in vitro* studies using cultured podocytes. We treated podocytes with HB-EGF and observed upregulation of p-EGFR and p-STAT3. We also observed HB-EGF-induced upregulation of p-EGFR and p-STAT3 was prevented by Dex (Figure 6A). However, the total protein levels of both EGFR and STAT3 in the cells were not changed (Supplementary Figure S2).

Notch signaling is activated in podocytes of glomerular diseases (27), and Notch3 has been further shown to be activated and promote crescent formation in glomerulonephritis in animal (28), We therefore examined Notch3 signaling in cultured podocytes treated with HB-EGF, and found it was activated as indicated by increased Notch3 intracellular domain (NICD3) (Figure 6B). We also found that the mRNA levels of key components of Notch signaling, including Notch1, Notch3, Hes1 and Hey1, was significantly increased in podocytes treated with HB-EGF, compared to controls (Figure 6C). However, the upregulation of the Notch signaling components was abolished by Dex (Figures 6B,C).

### Conditioned Medium From HB-EGF-Treated Podocytes Activated Notch Signaling and Enhanced Proliferative Activity in Primary PECs

**Figure 7:**
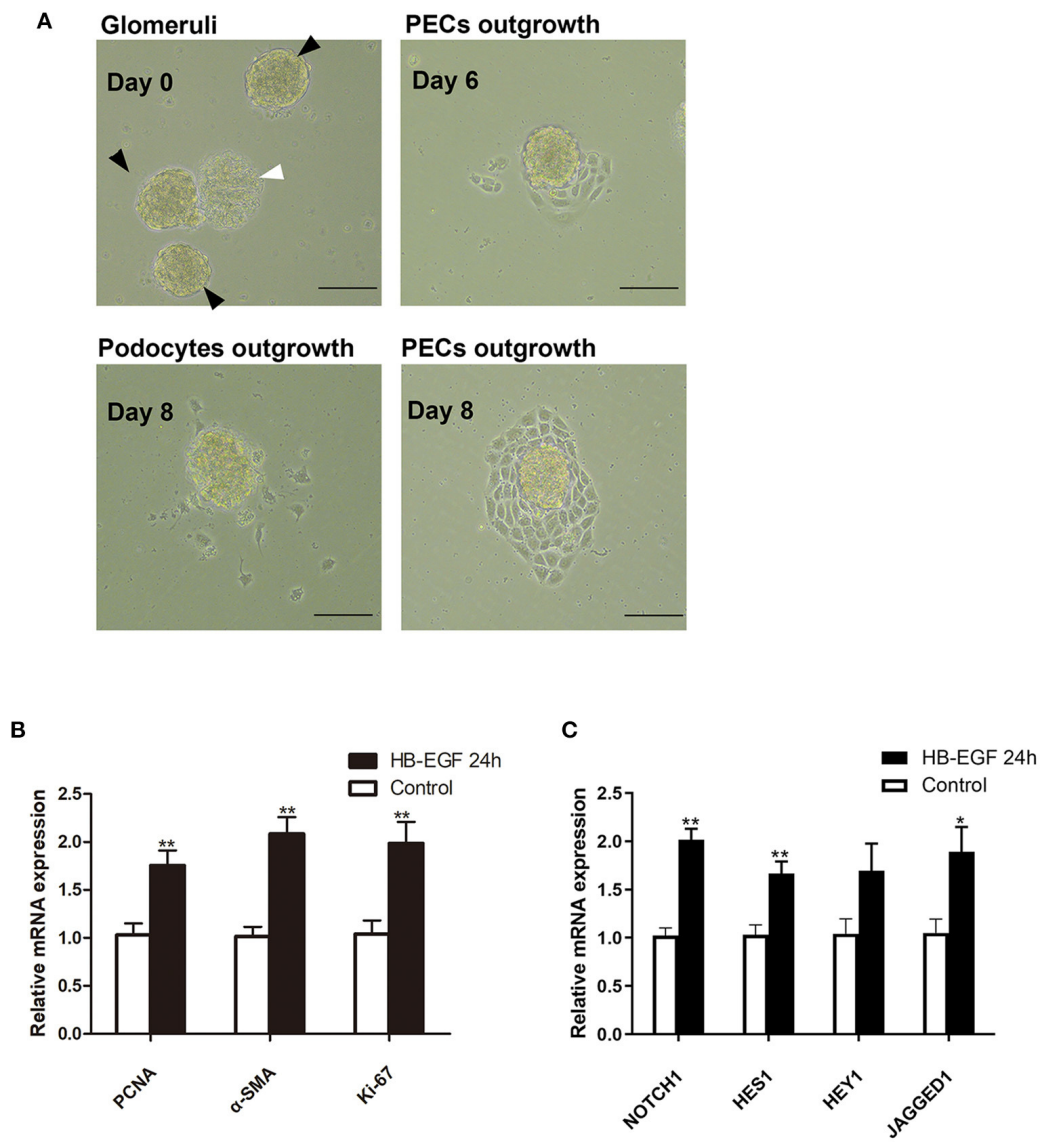
Conditioned medium from HB-EGF-treated podocytes activated Notch signaling and enhanced proliferative activity in primary PECs. **(A)** Capsulated (black arrows) and decapsulated (white arrow) glomeruli were isolated from rat kidney. Primary PECs (irregularly shaped, uniform in size, and grown in clusters) outgrowing from capsulated glomeruli at day 7 were seen. **(B)** qRT-PCR analysis showing that the relative PCNA, α-SMA, Ki-67 mRNA expressions were significantly upregulated in the primary PECs after 24-h stimulation with conditioned medium from HB-EGF-treated podocytes **(C)** qRT-PCR analysis of Notch and downstream genes revealed a marked increase of mRNA in the same PECs as in **(B)**. All data are presented as the mean ± SD of three independent experiments. *P < 0.05, **P < 0.01 vs. primary PECs treated with control medium from untreated podocytes. Scale bars, 50 μm.

According to recent studies, the most important role for podocytes in the formation of cellular crescent is to secrete soluble factors that act on PECs to promote their proliferation, other than dedifferentiate and proliferate to contribute to the crescents, under stimulation of immunocomplex or inflammatory factors. Based on the results described above, we speculated EGF signaling may mediate the expression of the secreted factors in podocytes. To prove this hypothesis, we treated podocytes with HB-EGF and used the medium to treat PECs. As shown in Figure 7A, we established successfully the culture of primary PECs derived from isolated rat glomeruli (capsulated). The cells were treated with conditioned medium from cultured podocytes treated with HB-EGF for 24 h to test the medium for presence of factors capable of promoting proliferation of PECs. We found that mRNA expression of markers for cell proliferation (Ki-67, PCNA) and trans-differentiation (α-SMA) were significantly upregulated in the primary PECs after 24 h stimulation using the conditioned medium, compared to the controls (Figure 7B). Besides, Notch signaling was also activated in the cells (Figure 7C).

### Glucocorticoids Inhibited Expression of EGFR Ligands *in vivo* and *in vitro*

**Figure 8:**
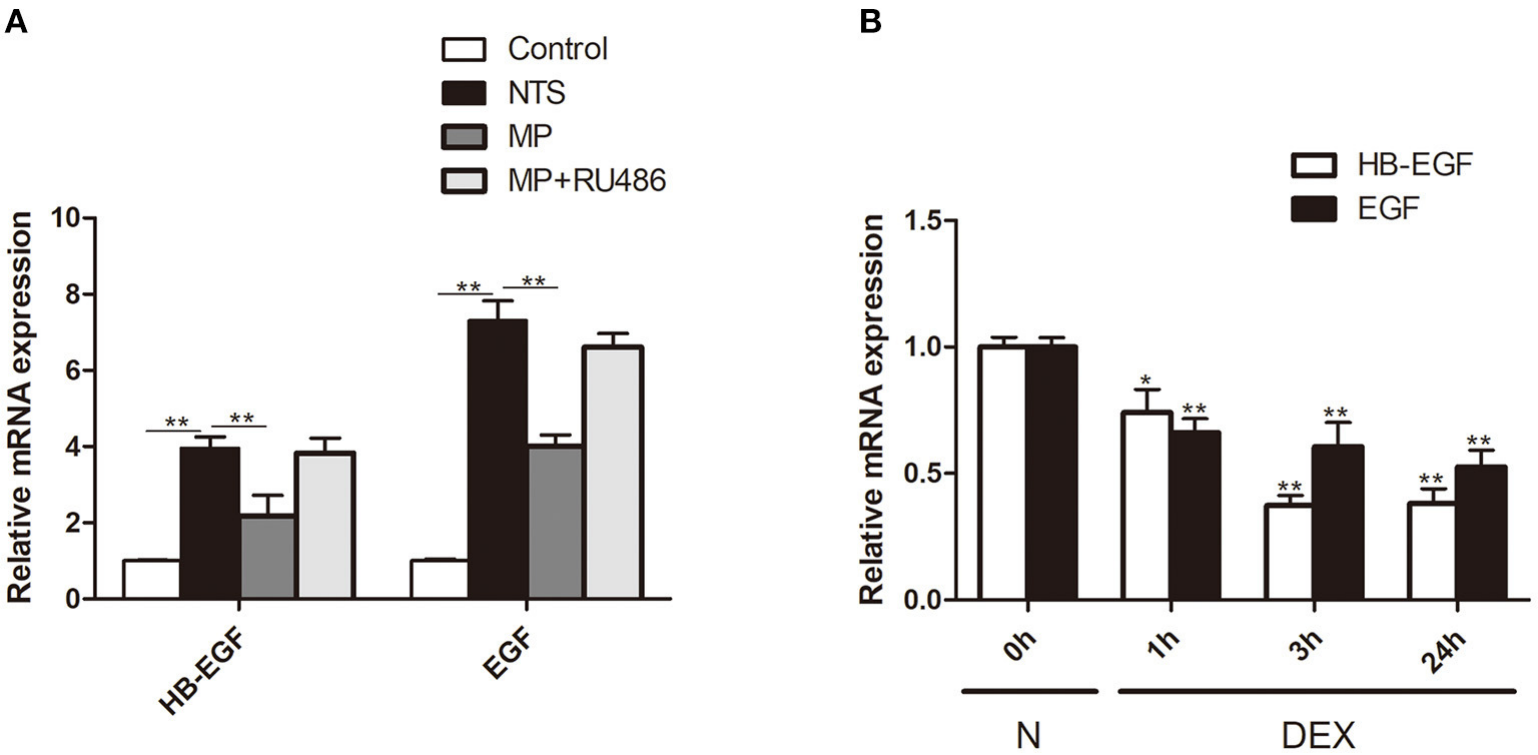
Glucocorticoid treatment inhibited expression of EGFR ligands *in vivo* and *in vitro*. **(A)** Quantification by qRT-PCR of HB-EGF and EGF mRNA in isolated glomeruli of rats treated with saline (control), NTS, NTS+MP, NTS+MP+RU486, respectively on day 14 (*n* = 5 per group). **P < 0.01; **(B)** qRT-PCR analysis showing the relative HB-EGF and EGF mRNA expression levels in cultured podocytes stimulated with Dex (1 μM) for 0, 1, 3, 24 h, respectively, *in vitro*. The data are expressed as the mean ± SD of three independent experiments. *P < 0.05, **P < 0.01 vs. mRNA levels on 0 h at baseline.

To explore the mechanism underlying glucocorticoids' inhibition of EGF signaling, we tested the effect of glucocorticoids on the expression of HB-EGF, an EGFR ligand that is known to be *de novo* induced in patients with RPGN but not non crescentic glomerulonephritis (18). We observed that the mRNA expression of HB-EGF, as well as another EGFR ligand EGF, was markedly upregulated in glomeruli after induction of NTS nephritis (Figure 8A), which was in parallel with activation of EGFR/STAT3 signaling (Figure 4). MP treatment significantly inhibited the upregulation of mRNA of HB-EGF and EGF (Figure 8A). *In vitro*, we found that the relative mRNA expression of HB-EGF and EGF were both significantly downregulated in cultured podocytes treated with Dex. These observations demonstrated that glucocorticoids exert its effect through inhibition of EGF ligand expression (Figure 8B).

### Glucocorticoids Induced Expression of EGFR Inhibitor Gene 33

EGF signaling is initiated by autophosphorylation of EGFR tyrosine kinase. Activated EGFR can be immediately targeted by the inhibitory mechanisms, including binding and inhibition by Gene 33 (ERRFI1), a negative feedback regulator of EGFR. We therefore examined the effect of glucocorticoids on Gene 33 expression in glomeruli of rats treated with NTS, and found that Gene 33 mRNA was increased by >5 times compared with control rats, suggesting an autonomous negative feedback on EGFR activation. The mRNA level of Gene 33 was further elevated after administration of MP, and the GR antagonist abolished the effect of MP (Supplementary Figure S3A), suggesting that glucocorticoids inhibit EGFR activation by upregulating Gene 33.

We also examined the effect of glucocorticoids on the expression of Gene 33 in cultured podocytes, and found that Gene 33 mRNA was also increased by HB-EGF but to an extent much less than that *in vivo* (Supplementary Figure S3B), and further increased by Dex. Consistently, Gene 33 protein was also increased by HB-EGF and further increased by Dex (Supplementary Figure S3C).

## Discussion

Anti–GBM glomerulonephritis is a form of rapidly progressive glomerulonephritis (RPGN), characterized by rapid progression, poor prognosis, and massive crescentic formation in glomeruli. As a routine and effective treatment for anti-GBM crescentic glomerulonephritis, the mechanisms underlying effectiveness of glucocorticoids have not been carefully determined. Understanding the mechanisms would likely provide improved therapeutic strategies for the disease.

Consistently with our previous studies with anti-GBM patients (21), we found that PECs and activated PECs were also increased in glomerular crescents of rat model of anti-GBM glomerulonephritis. This observation supports a key role of PECs in crescent formation. Thus, it is crucial to elucidate which signaling triggers the activation and proliferation of PECs in order to better understand pathogenesis of the disease.

Previous studies have suggested multiple mechanisms underlying PEC activation and proliferation, including the one that stimulated podocytes secrete certain factors that promote PEC activation and proliferation (14), as suggested by the observations that EGFR or STAT3 deletion in podocytes ameliorates PEC proliferation and crescent formation (18, 19). Podocyte EGFR/STAT3 signaling may turn on the expression of secreted factors that promote PEC activation and proliferation. We did observe the activation of EGFR/STAT3 signaling in podocytes, which was accompanied by activation and proliferation of PECs, in both anti-GBM patients and rat model. In the treatment of glucocorticoids the activation of EGFR/STAT3 was abolished. This result suggests that glucocorticoids exerts therapeutic effect on the disease at least partly by preventing activation of EGFR/STAT3 pathway.

Notch signaling, as an evolutionarily conserved intercellular signaling pathway which regulates interactions between physically adjacent cells, is involved in kidney development, but its activity is rarely detected in fully developed glomeruli (29). It has been shown that Notch signaling in podocytes is required for PEC proliferation and the development of anti-GBM crescentic glomerulonephritis (28). We also observed Notch1 activation in podocytes of the NTS-treated rats, which was abolished by glucocorticoids treatment (Figure 5). These studies suggest a link between EGFR/STAT3 and Notch signaling in the disease. Indeed, we observed that HB-EGF was capable of inducing Notch signaling activation in cultured podocytes, indicating that Notch signaling is downstream of EGFR/STAT3. Supportively, previous studies have shown that STAT3 can directly bind to the promoter of Notch ligands, including Jagged1 and Dll1, to induce their expression (30, 31).

Le Hir et al. have demonstrated podocyte bridges between the glomerular tuft and Bowman's capsule, an early event in crescent formation (10). Given that Notch signaling is dependent on direct contact between ligand-expressing cells and receptor-expressing cells, and that STAT3 can directly bind to the promoter of Notch ligands, Jagged1 and Dll1, to induce their expression (30, 31), we may propose a new model for PEC activation and proliferation, that is, a stimulus (e.g., NTS) induces EGFR/STAT3 activation in podocytes; then the activated STAT3 in turn drives Jagged1/Dll1 expression; next, the Jagged1/Dll1 are presented to the Notch receptors on PECs through podocyte bridges to activate Notch signaling in PECs, resulting in PEC activation and proliferation, and eventually crescent formation. With the treatment of glucocorticoids the entire pathway is blocked from the beginning.

To prove the speculation that podocytes with activated EGF signaling could secrete factors capable of activating PECs, we treated cultured podocytes with HB-EGF and collected the conditioned medium to treat primary PECs. The conditioned medium was found to activate Notch signaling and enhance proliferative activity in the PECs (Figure 7). These results were consistent with previous finding that Notch signaling mediates PEC activation and proliferation (28). Studies have shown that induction of Notch signaling increased the expression of mesenchymal phenotypic genes, including E-cadherin, α-SMA, vimentin, and Snail, in PECs, and promoted PEC hyperplasia and migration (32). It appears that the development of crescentic glomerulonephritis may also follow the sequence of EGFR/STAT3/Notch activation, secreted factors expression in podocytes, and Notch activation and cellular proliferation in PECs.

Glucocorticoids are known to exert therapeutic effect by genomic and various non-genomic mechanisms. The canonical genomic mechanism involves GR, thus, RU486, an antagonist of GR, can be used to determine whether an effect of glucocorticoids is through GR-dependent genomic mechanism. We showed that glucocorticoids markedly attenuated proteinuria, serum creatinine elevation, crescent formation; in addition, it effectively suppressed the activation and proliferation of PECs and other clinical and pathological worsening. Our results were consistent with other studies demonstrating podocytes are direct target of glucocorticoids (33–38). In a recent study by Zhou and colleagues, specific deletion of GR gene in podocytes was found to worsen podocyte injury after NTS treatment; however, the percentage of crescentic glomeruli was similar in the control and knockout mice (39).

To explore how glucocorticoids has inhibitory effect on EGFR activity, we performed studies in cultured podocytes. We found that glucocorticoids can inhibit expression of EGF ligands (HB-EGF, EGF), and prevent activation of EGFR, STAT3 and Notch3. These effects of glucocorticoids were abolished by RU486, suggesting that glucocorticoids inhibitory effect is through its receptor, GR, in podocytes (Figure 8). Apparently, expression inhibition of EGF ligands could underlie the entire effects of glucocorticoids. Furthermore, we investigated whether glucocorticoids have any effect on the expression of Gene 33, a feedback inhibitor of EGFR activation. Previous studies have shown that Gene-33 can be upregulated by EGF, HB-EGF, platelet-derived growth factor, serum, and dexamethasone (40, 41). Consistently, we also found that glucocorticoids upregulated Gene 33 at both mRNA and protein levels in cultured podocytes (Supplementary Figure S3), which likely contributes to glucocorticoids-induced inhibition of EGFR and the downstream pathway. Thus, upregulation of Gene 33 may represent an additional mechanism underlying inhibitory effect of glucocorticoids on EGFR activation.

There are several limitations in the present study. Firstly, we had limited number of control groups in the animal study (Figure 3) and lacked the groups of MP alone, RU486 alone, and NTS+RU486. According to previous relevant studies, MP or RU486 alone does not cause overt renal changes in normal animals. However, RU486 has been shown to be protective and alleviate NTS-induced crescent formation *via* a complicate mechanism (42). We therefore did not include the NTS+RU486 group as control in order to simplify the experiments. Nevertheless, we believe that lack of the NTS+RU486 control did not affect data interpretation because the NTS+MP+RU486 group exhibited more severe injury compared with the NTS+MP group, a result that was in contrast with RU486 protective effect in the NTS-treated animals, suggesting that RU486 antagonized MP to reduce its therapeutic effectiveness.

Secondly, there was lack of co-staining of p-EGFR/pSTAT3 with podocyte markers to confirm the activation of EGFR and STAT3 specifically in podocytes. Since the cells at the periphery of a glomerulus are usually podocytes, we focused on this population of podocytes for p-EGFR and p-STAT3 localization. The results showed that p-EGFR and p-STAT3 was specifically induced in podocytes in both anti-GBM patients and NTS-treated rats, consistent with the studies by others (18, 19).

Thirdly, there might be an interference of immune cells on the results because immune cells are also responsive to glucocorticoids to reduce their injurious activity, thereby contributing to the alleviation of disease in the NTS-treated rats. Ideally, Rats with immune-cell specific GR deletion are used to exclude the influence of immune cells on the results. Nevertheless, the conclusions drawn from the present study should be still reliable because we focused on glucocorticoids effect on EGFR/STAT3 signaling which is known to contribute to glomerular crescent formation in NTS-treated animals; besides we did observe inhibitory effect of glucocorticoids on EGFR/STAT3 signaling activation. Moreover, this study also focused on the early event of crescent formation, i.e., activation of podocytes and PECs, when immune cells are blocked by intact Bowman's capsule, thus unable to enter glomeruli to play their role in crescent formation (43).

Finally, we did not perform study with podocyte-specific GR knockout mice. Zhou and colleagues made NTS model using podocyte-specific GR knockout mice and found that crescent formation was similar between the knockout and control mice (39). Unfortunately, they did not treat the mice with glucocorticoids and examined whether there was any difference in PEC phenotype and crescent formation between the knockout and control mice after NTS treatment. We would expect that glucocorticoids would have reduced therapeutic effectiveness on NTS-treated knockout mice compared with wild-type mice.

**Figure 9:**
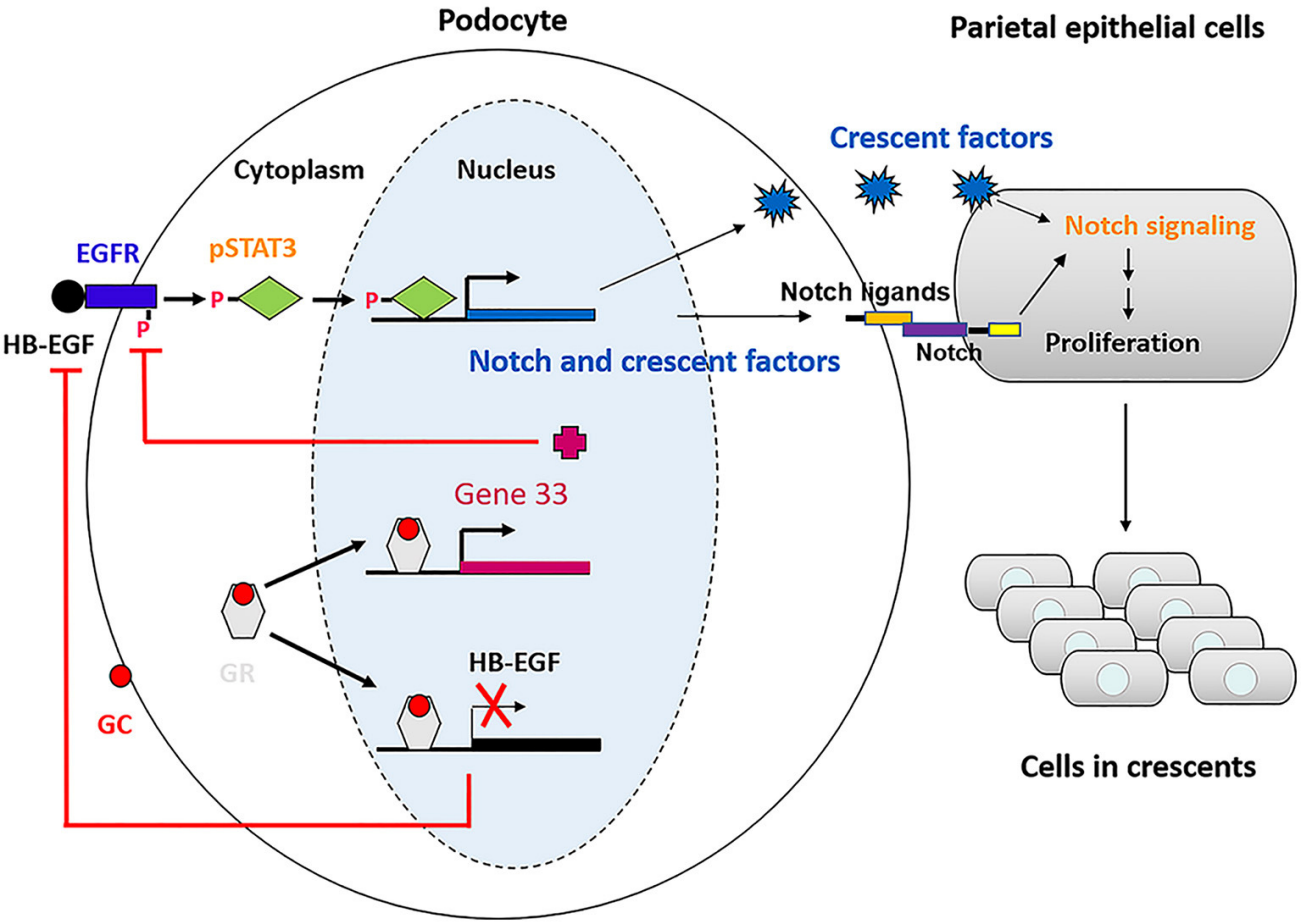
Schematic of the mechanism underlying glucocorticoids effectiveness on anti-GBM crescentic glomerulonephritis.

Based on our studies and previous studies by others, we propose a model illustrating the mechanism underlying therapeutic effectiveness of glucocorticoids on anti-GBM crescentic glomerulonephritis (Figure 9). This proposed model of mechanism may be helpful for treatment of the disease in which glucocorticoids are used.

## Conclusion

In conclusion, aberrant activation of EGF signaling in podocytes might be a key step in crescent formation and RPGN progression. Glucocorticoids may suppress expression of EGFR ligands and upregulate expression of Gene 33, thereby preventing activation of EGFR and downstream pathway that leads to PEC activation and proliferation, and eventually alleviating anti-GBM crescentic glomerulonephritis. Further study may be required to fully prove this novel mechanism, hopefully providing new therapeutic strategies for the disease.

## Data Availability Statement

The original contributions presented in the study are included in the article/Supplementary Material, further inquiries can be directed to the corresponding author/s.

## Ethics Statement

The studies involving human participants were reviewed and approved by the Human Subjects Committee of Jinling Hospital. The patients/participants provided their written informed consent to participate in this study. The animal study was reviewed and approved by the Institute Animal Care and Use Committee of Jingling Hospital.

## Author Contributions

ZT, SS, JZ, and XW designed the study. XW and LR performed experiments and interpreted data. QY, HS, and QT performed experiments. XW, ZT, JZ, and SS wrote the manuscript. MZ provided technical support. All the authors approved the manuscript.

## Funding

This work was supported by the Social Development Project of Jiangsu Province (BE2020698), the National Key R&D Program of China (Grant No. 2018YFC1312705), and the National Natural Science Foundation of China (Grant No. 81970619).

## Conflict of Interest

The authors declare that the research was conducted in the absence of any commercial or financial relationships that could be construed as a potential conflict of interest.

## Publisher's Note

All claims expressed in this article are solely those of the authors and do not necessarily represent those of their affiliated organizations, or those of the publisher, the editors and the reviewers. Any product that may be evaluated in this article, or claim that may be made by its manufacturer, is not guaranteed or endorsed by the publisher.
