# Supplementary figures and images for: Glucocorticoids Inhibit EGFR Signaling Activation in Podocytes in Anti-GBM Crescentic Glomerulonephritis
Source: Front Med (Lausanne). 2022 Feb 10;9:697443. doi: 10.3389/fmed.2022.697443 (PMC8866651; doi:10.3389/fmed.2022.697443)

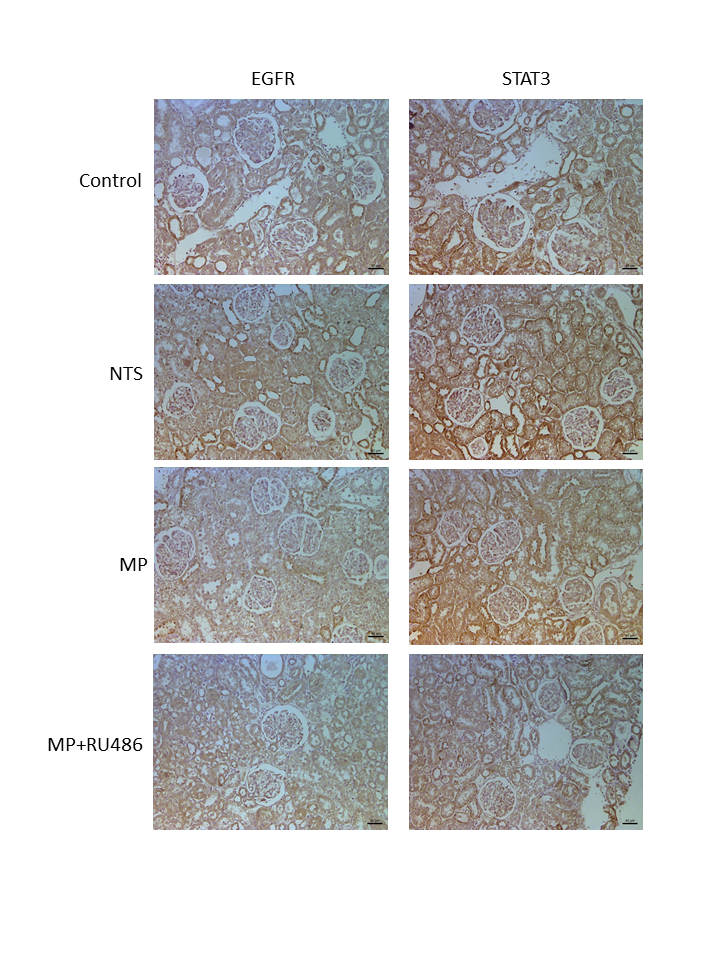

Supplement: Supplementary file 1 [file Image_1.TIF]

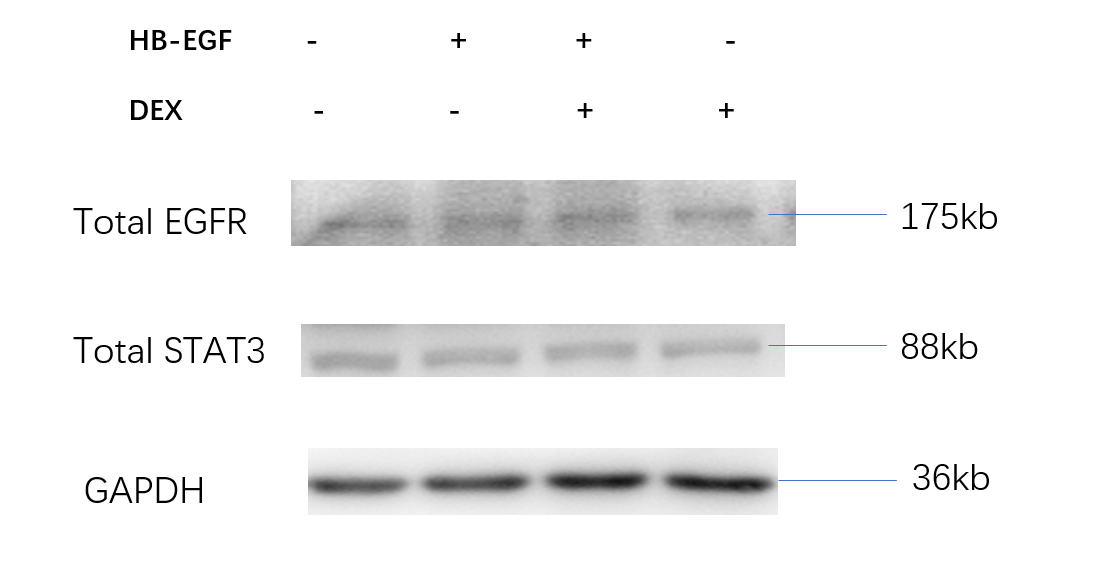

Supplement: Supplementary file 2 [file Image_2.TIF]

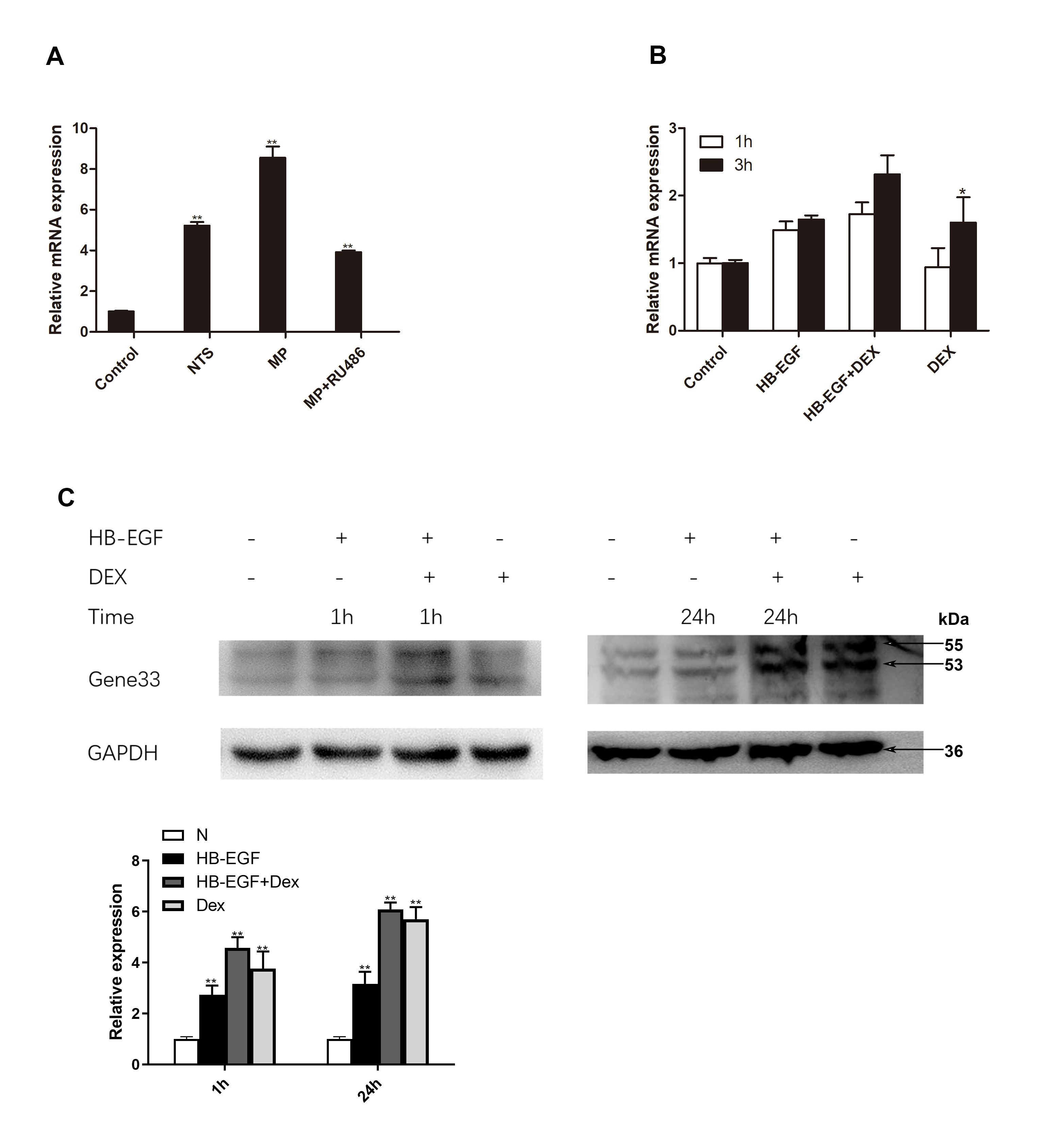

Supplement: Supplementary file 3 [file Image_3.TIF]

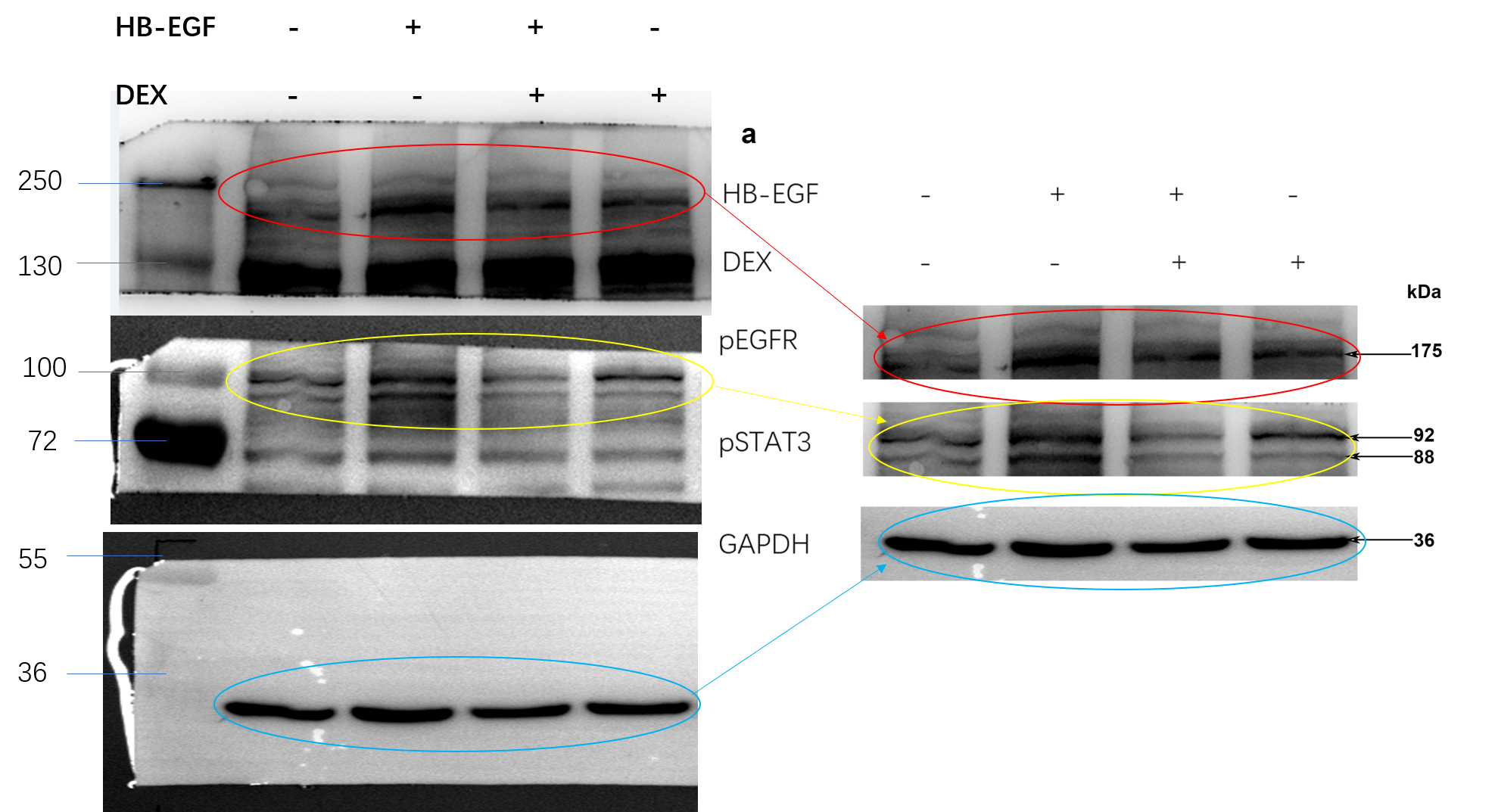

Supplement: Supplementary file 4 [file Data_Sheet_1.ZIP › Full uncropped and unedited versions of the Western blots for Figure 6a.tif]

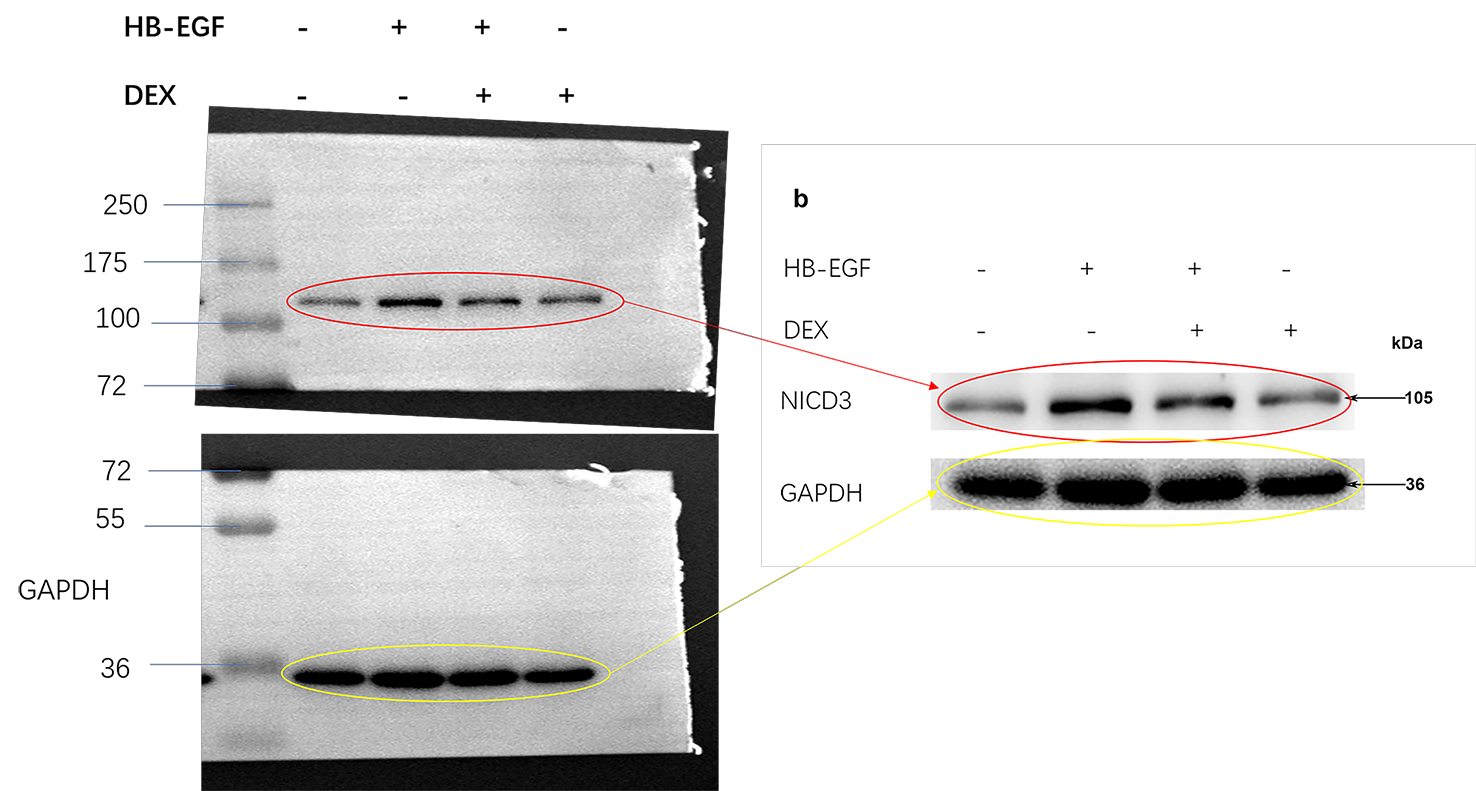

Supplement: Supplementary file 4 [file Data_Sheet_1.ZIP › Full uncropped and unedited versions of the Western blots for Figure 6b.tif]

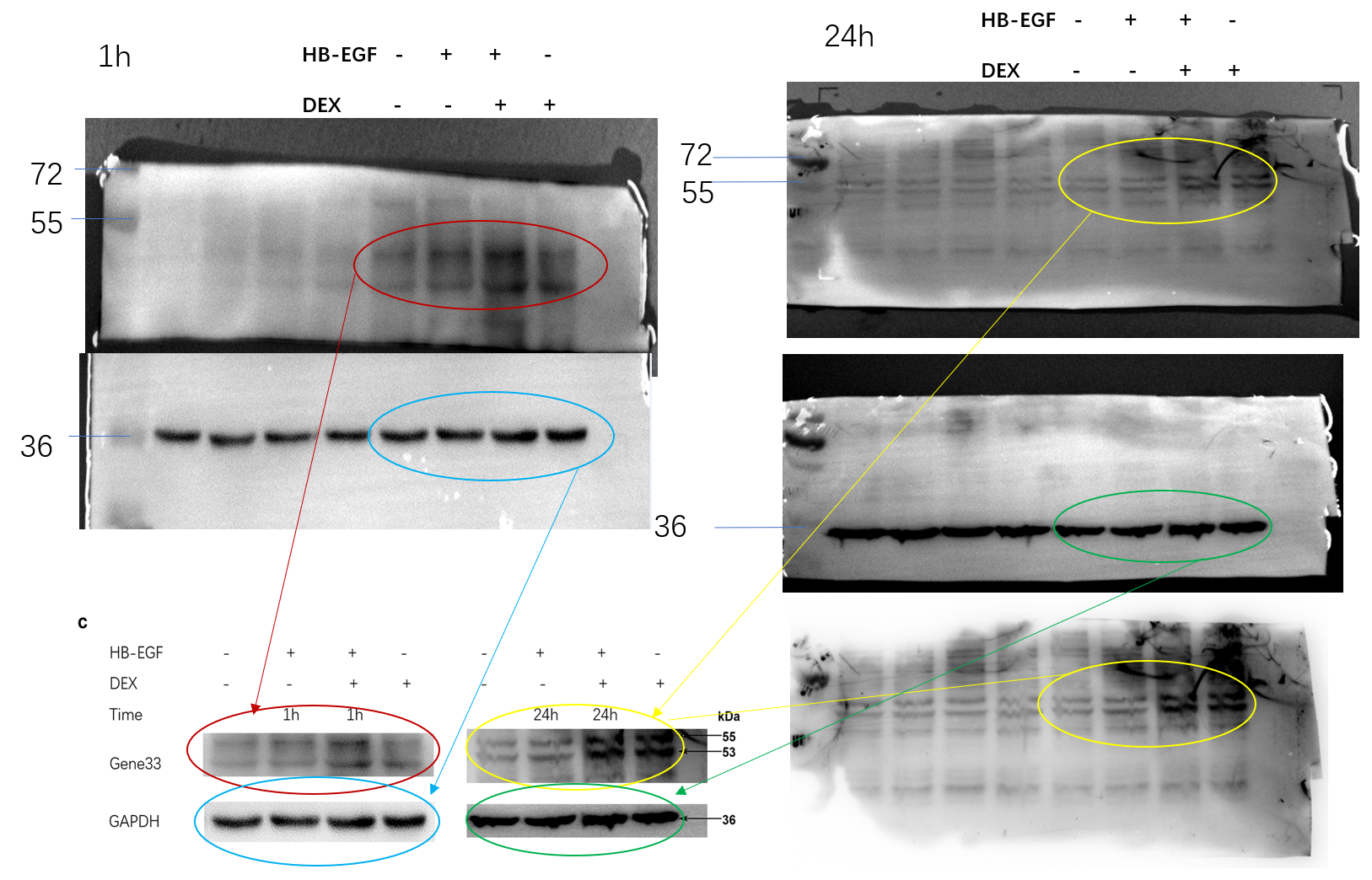

Supplement: Supplementary file 4 [file Data_Sheet_1.ZIP › Full uncropped and unedited versions of the Western blots for Supplementary Figure S1c..tif]
