# Supplementary material for: Glucocorticoids Inhibit EGFR Signaling Activation in Podocytes in Anti-GBM Crescentic Glomerulonephritis
Source: Front Med (Lausanne). 2022 Feb 10;9:697443. doi: 10.3389/fmed.2022.697443 (PMC8866651; doi:10.3389/fmed.2022.697443)
Supplement: Supplementary file 5 [file Table_1.docx]

**Supplementary information**

Glucocorticoids inhibit EGFR signaling activation in podocytes in anti-GBM crescentic glomerulonephritis

Xiaomei Wu, Lu Ren, Qianqian Yang, Hui Song, Qiaoli Tang, Mingchao Zhang, Jiong Zhang, Zheng Tang, Shaolin Shi

Supplementary Figure S1. Total EGFR and STAT3 protein levels were not changed in glomeruli of the NTS rats and neither affected by glucocorticoids treatment. Immunohistochemical staining of total EGFR and STAT3 protein in kidney of the rats treated with NTS and/or methylprednisolone.

Supplementary Figure S2. Immunoblotting of total EGFR and STAT3 protein in cultured podocytes showed that HB-EGF and Dex did not affect the protein levels of EGFR and STAT3 in cultured podocytes.

Supplementary Figure S3. Glucocorticoids upregulated Gene 33 expression. (A) qRT-PCR analyses showing the relative mRNA expression of Gene 33 from isolated glomeruli of the rats treated with saline (control), NTS, NTS+MP, NTS+MP+RU486, respectively on day 14. *P <0.05, **P <0.01 versus controls at baseline; (B) Quantification by qRT-PCR of Gene 33 mRNA in podocytes after stimulation by HB-EGF, HB-EGF+Dex, and Dex only for 1 hour and 3 hours, respectively. (C) Immunoblotting of Gene 33 in podocytes showing the expression of Gene 33 protein was upregulated by HB-EGF treatment for 1 hour and 24 hours, and further increased in the presence of Dex. Dex treatment alone also upregulated Gene 33. Quantifications of the blots are shown on the right. All data are presented as the mean±SD of three independent experiments. *P <0.05, **P <0.01 versus untreated control;

**Supplementary Table 1**. Primers and their sequences.

| Gene | | 5’- 3’ |
| --- | --- | --- |
| r-Notch1 | Primer F | CCGGCAGTGTGTACAGTCAT |
|  | Primer R | ACCGTCTCACTCTTTACGGC |
| r-Hes-1 | Primer F | CTACCCCAGCCAGTGTCAAC |
|  | Primer R | ATGCCGGGAGCTATCTTTCT |
| r-Hey-1 | Primer F | GGACTATCGGAGTTTGGGGTTT |
|  | Primer R | AGTTGTTGAGATGGGAGACCAG |
| r-Jagged | Primer F | GATGTCTCCAGGTCTCACCACT |
|  | Primer R | GATAAGGAAGGCTCACAGGCTA |
| r-PCNA | Primer F | GAAGGCTTCGACACATACCG |
|  | Primer R | TTTTGGACATGCTGGTGAGGT |
| r-Ki-67 | Primer F | ACTTGCCTCCTAATACTCCACTCA |
|  | Primer R | ATCTTCGTCTTTCATCATTTGTCC |
| r-α-SMA | Primer F | CTATGCTCTGCCTCATGCCA |
|  | Primer R | CTCACGCTCAGCAGTAGTCA |
| r-Notch3 | Primer F | GGCAGATGGCTCTACTGCAT |
|  | Primer R | CGATTTCCCGAGCTCATCCA |
| h- Notch1 | Primer F | ATAGTCTGCCACGCCTCTG |
|  | Primer R | AGTGTGAAGCGGCCAATG |
| h-Hes 1 | Primer F | AGCACACTTGGGTCTGTGC |
|  | Primer R | TGAAGAAAGATAGCTCGCGG |
| h-Hey 1 | Primer F | AGATAACGCGCAACTTCTGC |
|  | Primer R | GAGATCCTGCAGATGACCGT |
| 18s RNA | Primer F | TTTCTCGATTCCGTGGGTGG |
|  | Primer R | AGCATGCCAGAGTCTCGTTC |
| GAPDH | Primer F | GGCACAGTCAAGGCTGAGAATG |
|  | Primer R | ATGGTGGTGAAGACGCCAGTA |
